# Flow-Cytometry in the Diagnosis of Diffuse Large B-Cell Lymphoma Based on Stomach Tissue Samples: A Case Report

**DOI:** 10.7759/cureus.21766

**Published:** 2022-01-31

**Authors:** Tung Tuan Nguyen, An Thi Vinh Do, Nga Thi Nguyen, Thanh Quoc Truong, Ai Thuc Ton

**Affiliations:** 1 Hematology and Blood Transfusion, Bach Mai Hospital, Hanoi, VNM; 2 Hematology, Hanoi Medical University, Hanoi, VNM; 3 Pathology, Bach Mai Hospital, Hanoi, VNM

**Keywords:** lymphoma, immunohistochemistry, flow cytometry, fresh tissue, diffuse large b-cell lymphoma

## Abstract

Morphology and immunohistochemistry on node, tissue, and bone marrow biopsies are frequently used in lymphoma diagnosis to characterize the stage and subtype of diseases. Multicolor flow cytometry technology is a novel technique for the analysis of immunological markers to identify lymphoma on fresh tissue when immunohistochemical staining is ambiguous. We report a case of a patient diagnosed with diffuse large B-cell lymphoma by flow cytometry on a stomach tissue biopsy.

## Introduction

Diffuse large B-cell lymphoma (DLBCL) is a type of non-Hodgkin lymphoma, which is reported with relatively high frequency in patients with Epstein-Barr virus (EBV) infections, accounting for around 10% of all newly diagnosed lymphomas in Asia [1-3]. In addition, up to 40% of cases of DLBCL were detected in the gastrointestinal (GI) systems and were not associated with other GI tract malignancies [4]. Cases of lymphoid proliferation in the marrow were reported in 10-25% of the total DLBCL, with the presence of polymorphic large lymphocytes [5, 6]. Rich T-lymphocyte DLBCL is a variant with centroblast lymphocytes containing special morphologies such as medium to large size, oval shape, possibly nuclei, and basophilic protoplasm [7]. The immunophenotype of T-cell rich DLBCL is defined by following markers including CD19+, CD20+, CD22+, CD79a+; CD5+ (5-10%); CD10+, MUM1+ (30-50%); BCL6+ (60-90%) [8]. The morphology and immunohistochemistry (IHC) of DLBCL patients' bone marrow and lymph nodes show unusual T-lymphocyte proliferation, making diagnosis challenging. Tsagarakis et al. demonstrated tissue flow cytometry (FC) in conjunction with IHC to diagnose earlier lymphoma patients, particularly in the gastrointestinal system [9].

## Case presentation

**Figure 1:**
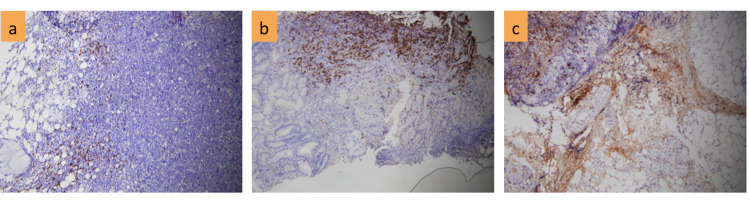
IHC of CD20+ in bone marrow (a); stomach(b); node(c)

**Figure 2:**
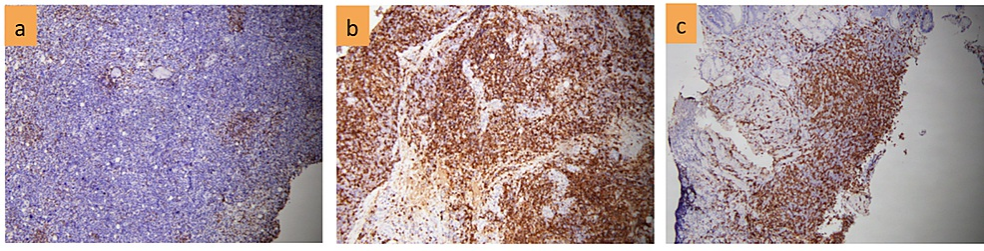
IHC of CD3+ in bone marrow(a); node(b); stomach(c)

**Figure 3:**
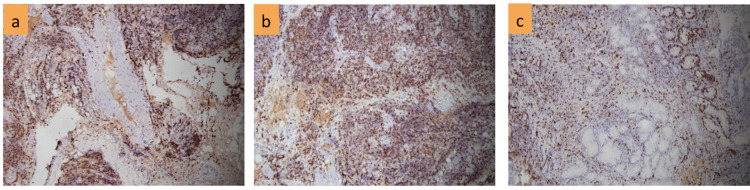
IHC of Ki67+ in bone marrow(a); node(b); stomach(c)

A 58-year-old male patient with a normal medical history was admitted to the hospital with fever (39 degrees). He coughed and spat phlegm, and had anemia, numerous lymph nodes in the neck, crackles in both lungs, mild hepatomegaly, and splenomegaly. Complete blood count showed severe anemia (Hb 70 g/l), leukocytosis (WBC 28.9 x 10^9^/L) with 68% of neutrophils and normal range of platelets; elevated D-dimer (7,421 mg/l FEU); positive direct and indirect Coombs test. Liver functions were abnormal with increasing AST (89 U/L), ALT (91 U/L), and low albumin level (16.5 g/l); bilirubin level, calcium level, and renal function were all normal. Inflammatory markers include high levels of lactate (3.9 mmol/l), hs-CRP (12.1 mg/dl), and LDH (780 U/l). Aspiration of bone marrow revealed a variety of morphological abnormalities, including small to medium-sized lymphocytes with deformed and faceted nuclei, as well as giant lymphocytes with large nuclei as lymphocytic activity. Bone marrow biopsy showed that the density of cells was enhanced with erythroid and myeloid hyperplasia, as well as slight megakaryocytic lineage suppression. Lymphoid proliferation overwhelmed other cells in some areas, primarily tiny lymphocytes with deformed nuclei. IHC staining of bone marrow biopsy samples indicated reactive lymphocytosis. The karyotype was normal (46,XY). The result of cervical lymph node biopsy was resolved as structural lymphadenitis with total lymphatic system deletion, atypical lymphoproliferative, CD20 positive for B cells and CD3 positive for T cells, Ki67+ 80%, MUM1+; negative for CD23, CD10, Bcl6 (Figures 1-3). Endoscopy showed an ulcer of the stomach. A considerable number of large nuclei, hyperchromatic, cytoplasmic cells, and a high multiplication ratio were seen in the biopsy samples.

**Figure 4:**
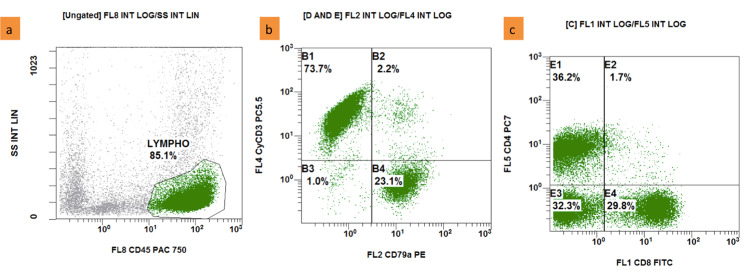
Flow cytometry on fresh tissue from stomach biopsy (a): Lymphocytes (green dots) accounted for 85,1%; (b): Lymphocytes (green dots) positive for cyCD3 (73,7%), positive for CD79a (23,1%); (c): positive for CD4, CD8 (Navios EX - Beckman Coulter)

**Figure 5:**
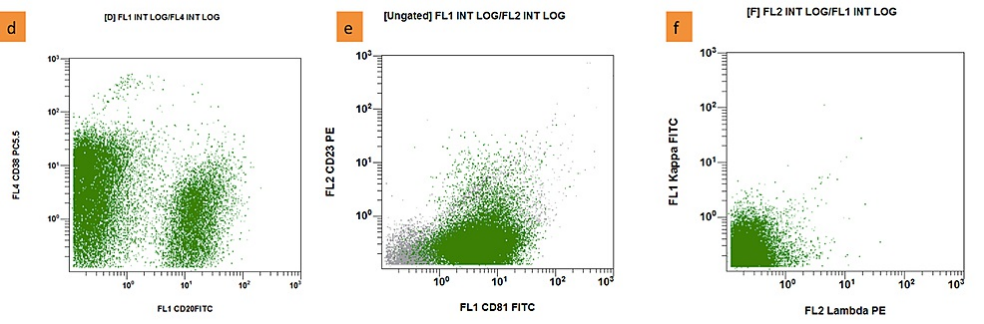
Flow cytometry on fresh tissue from stomach biopsy samples (d): Lymphocytes (green dots) positive for CD20, CD38; (e): positive for CD81, negative for CD23; (f): negative for kappa, lambda (Navios EX - Beckman Coulter)

Flow cytometry of fresh tissue from gastric biopsies samples revealed a high proportion of lymphocytes (approximately 85%), with T lymphocytes accounting for the majority (73%) with the immunophenotype of mature T lymphocytes: positive for CD2, CD3, CyCD3, CD5, CD7, CD4 or CD8 (CD4:CD8 ratio is approximately 1.4:1). B lymphocytes, which accounted for a lower proportion (23%), were positive for CD19, CD20, CD22, CD79a, CD81, weakly positive for FMC7, and negative for CD10, CD11c, CD103, kappa, and lambda (Figure 4, 5). CD4/CD8 expression in fresh tissue pieces was normal. Although B cells were scattered in a limited number, the inclusion of CD19, CD22, and CD79a markers alongside CD81, as well as the lack of light chain expression, has prompted us to reconsider B-cell lymphoma.

Combining with IHC staining results of gastric biopsy samples, in which tumor cells were positive for markers CD20, Ki67 (80%), MUM1, CD3, CD15, CD30, and EBV but negative for CD10, Bcl6, Bcl2, we determined T-cell/histiocyte-rich large B-cell lymphoma with EBV positivity in the stomach.

The patient was given antibiotics due to pneumonia. After that, he was treated with the R-CHOP regimen and continued to be monitored. The patient's fever was gone after the first course of treatment, and the liver and spleen were no longer palpable, as were the peripheral lymph nodes. Recent blood test showed Hb 81g/l, WBC 5.7 x 10^9^/L with 53% of neutrophils, and PLT 210 x 10^9^/L. The patient was monitored continuously in the outpatient department.

## Discussion

T cell/histiocyte-rich large BCL is an uncommon type of DLBCL that presents with a more malignant appearance and a poor prognosis. The liver, spleen, and bone marrow are all commonly affected by the disease, which is classed as stage III and stage IV (Ann-Arbor) at the moment of diagnosis [10]. Normal morphological, histochemical, and immunological traits are prevalent, with a few B cells (typically less than 10%) on an overwhelming T-lymphocyte background, causing numerous challenges in diagnosis, or easily misdiagnosing as T-cell lymphoma. IHC has long been regarded as the gold standard for lymphoma diagnosis. However, tissue immobilization can result in loss of some surface antigens, allowing analysis of roughly 100 cells and only one antigen per slide. The improved tissue flow cytometry allows for the examination of numerous antigens on a large number of cells (up to 100,000 cells) with a small tissue sample (0.1cm2 or less) in a short amount of time [11, 12].

We performed histopathological and immunohistochemistry staining of gastric biopsy samples during the diagnostic process. The histopathological image revealed a massive proliferation of large nuclear cells with narrow cytoplasm; positive for CD19 and CD20 markers, as well as positive EBV on a T-lymphocyte background. We determined that the T-lymphocyte population was high but were mainly mature T-lymphocytes with a CD4:CD8 ratio of approximately 1.4:1; whereas the B-lymphocyte population was abnormal with loss of expression of kappa and lambda, and increased expression of the CD81 marker on tissues flow cytometry. Reactive lymphocytosis may be related to the EBV virus, particularly T-CD8 cells (the ratio CD4:CD8 should not exceed 1:3); nonetheless, some investigations have found an increase in T-CD4 lymphocytes [13]. According to Luo et al., CD81 expression is thought to be a sign of DLBCL [14]. Glynn et al. discovered that T-cell histiocyte-rich large B cell lymphoma would diminish or lose expression of surface immunoglobulins, which was consistent with the tumor cell immunophenotype in this case [15].

Tsagarakis et al. proposed the method of combination with IHC and fresh tissue. Flow cytometry was conducted for definitive diagnosis of tumor lymphocyte in three of his patients with clinical manifestations of fever of unknown cause, aggressive clinical course, and hemophagocytic syndrome; this is similar to our patient whose symptoms developed rapidly in 10 days with high fever [9]. In 2013, Demurtas et al. released a study on the efficacy of flow cytometry in identifying difficult-to-differentiate B cells by IHC such as follicular lymphoma or reactive lymphoma [12].

Thus, we have concluded that T-lymphocytes are abundant in cases of larger B-cell lymphoma. With the CD20+, R-CHOP therapy is the standard first-line treatment. Eventually, our patient's symptoms and examination improved, and he was discharged after one cycle of chemotherapy treatment.

## Conclusions

The combination of multicolor flow cytometry immunoassay with immunohistochemical staining has contributed to the speedy and accurate diagnosis of lymphoma. This is a valuable technique that should be used in regular diagnostic lymphoma procedures, especially in cases when histology and immunohistochemistry yield ambiguous or atypical results, as in described cases.
